# Synergistic Effects of Surface Coating and Bulk Doping in Ni‐Rich Lithium Nickel Cobalt Manganese Oxide Cathode Materials for High‐Energy Lithium Ion Batteries

**DOI:** 10.1002/cssc.202102220

**Published:** 2021-12-02

**Authors:** Friederike Reissig, Martin Alexander Lange, Lukas Haneke, Tobias Placke, Wolfgang G. Zeier, Martin Winter, Richard Schmuch, Aurora Gomez‐Martin

**Affiliations:** ^1^ Helmholtz Institute Münster, IEK-12 Forschungszentrum Jülich GmbH Corrensstr. 46 48149 Münster Germany; ^2^ Department of Chemistry Johannes Gutenberg University Mainz Duesbergweg 10–14 55128 Mainz Germany; ^3^ MEET Battery Research Center Institute of Physical Chemistry University of Münster Corrensstr. 46 48149 Münster Germany; ^4^ Institute of Physical Chemistry University of Münster Corrensstr. 30 48149 Münster Germany

**Keywords:** cathode materials, lithium ion batteries, Ni-rich cathodes, W-coating, Zr-doping

## Abstract

Ni‐rich layered oxide cathodes are promising candidates to satisfy the increasing energy demand of lithium‐ion batteries for automotive applications. Thermal and cycling stability issues originating from increasing Ni contents are addressed by mitigation strategies such as elemental bulk substitution (“doping”) and surface coating. Although both approaches separately benefit the cycling stability, there are only few reports investigating the combination of two of such approaches. Herein, the combination of Zr as common dopant in commercial materials with effective Li_2_WO_4_ and WO_3_ coatings was investigated with special focus on the impact of different material processing conditions on structural parameters and electrochemical performance in nickel‐cobalt‐manganese (NCM) || graphite cells. Results indicated that the Zr^4+^ dopant diffusing to the surface during annealing improved the electrochemical performance compared to samples without additional coatings. This work emphasizes the importance to not only investigate the effect of individual dopants or coatings but also the influences between both.

## Graphical abstract

**(I can't get no) satisfaction**: Ni‐rich nickel‐cobalt‐manganese (NCM)‐type layered oxide materials are promising candidates to satisfy the increasing energy demand of lithium‐ion batteries for automotive applications but have major drawbacks in terms of mechanical stability and cycling stability. Herein, the challenges of combining two mitigation strategies (i. e., Zr^4+^ doping and W^6+^ coating), their synergistic effects, and the importance of investigating heat‐treated references are reported.

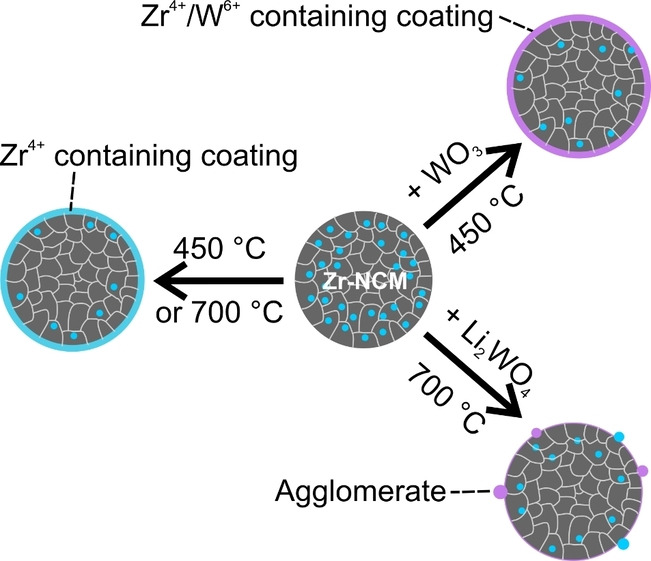

## Introduction

Considering the global climate crisis and the crucial need to reduce greenhouse gases, there is a huge demand for renewable energies. Innovations in different fields are necessary to account for the increased demand in energy generation, storage, and distribution. The storage of “green” electricity is one example with the challenge that every application has different requirements in cost, lifetime, power, and gravimetric and volumetric energy densities. In the sector of individual mobility, the customer will expect comparable cost, safety, and driving range of an electric vehicle (EV) compared to a vehicle powered by a combustion engine.^[1]^ Therefore, future generations of battery technologies in EVs need to become cheaper and at the same time provide higher energy density than today. Rechargeable lithium‐ion batteries (LIBs) are the technology of choice for electro‐mobility because of their high level of technological maturity combined with a good compromise between energy density, power, energy efficiency, lifetime, and costs.[^2^, ^3^, ^4^]

Ni‐rich LiNi_1‐*x*‐*y*_Co_*x*_Mn_*y*_O_2_ (NCM) layered oxide materials with Ni contents of 60–80 % are commercially available candidates for the positive electrode (cathode) to satisfy those needs and therefore enable extensive market penetration of EVs.[^5^, ^6^, ^7^] The main advantages of increasing the Ni content lie in an increased energy density on material level (higher de‐lithiation capacity at the same charge cut‐off potential) and a reduced demand for cobalt as critical raw material.^[8]^ There are still major challenges in terms of material stability and cycling stability for NCM materials with ≥80 % Ni,[^9^, ^10^] which include but are not limited to particle cracking, surface reconstruction^[11]^ to electrochemically inactive compounds, moisture sensitivity, self‐redox reactions, transition metal dissolution, parasitic reactions with the electrolyte, and, hence, the loss of active material upon cycling.[^12^, ^13^, ^14^, ^15^, ^16^]

Several mitigation strategies can be applied to improve cycle life, such as bulk cationic substitution (frequently called “doping”) to mitigate strong lattice parameter variations, coatings to protect the surface in contact with the electrolyte, and advanced particle design including core‐shell, concentration gradient particles, as well as single‐crystal particle approaches.[^12^, ^17^] Highly beneficial core‐shell and concentration gradient approaches in Ni‐rich layered cathodes have, for example, been extensively reported by Sun and co‐workers.[^18^, ^19^, ^20^] There is a broad range of cationic dopants that was investigated for Ni‐rich layered oxide cathode materials including, for example, Mg, Al, Ti, V, Mn, Fe, Co, Ga, Zr, and W.[^21^, ^22^, ^23^, ^24^, ^25^, ^26^, ^27^] Especially Zr as a high‐charge cation (Zr^4+^) has been extensively reported with various beneficial effects, such as forming a strong Zr−O bond to stabilize the layered structure or Zr^4+^ acting as pillar in the Li‐layer.[^28^, ^29^, ^30^] The variety of reported coatings is equally extensive, including organophosphates and metal oxides such as MgO, Al_2_O_3_, SiO_2_, La_2_O_3_, TiO_2_[^21^, ^31^, ^32^, ^33^, ^34^, ^35^] and various reports using ZrO_2_ or Li_6_Zr_2_O_7_.[^30^, ^36^] The working principle of those coatings is still poorly understood, but surface protection of the secondary particle by HF scavenging is one of the proposed mechanisms. In addition, WO_3_‐based coatings showed promising results in terms of improving cycle life and stabilizing the cathode/electrolyte interface of Ni‐rich cathodes.[^37^, ^38^] Unfortunately, previous studies either used expensive annealing gases (e. g., Ar)^[37]^ or lack long‐term cycling studies in realistic NCM || graphite full‐cells. Most reports are restricted to NCM || Li metal cells with an unlimited lithium inventory in the anode and have limited significance for the performance of resulting LIB full‐cells.^[39]^

Although it is well known that each of these modification approaches separately benefits the cycling stability of Ni‐rich cathodes, there are however limited systematic reports investigating the simultaneous combination of two of the approaches.^[12]^ To the best of our knowledge, the only reported simultaneous combinations of doping and coating are limited to the use of the same element for both, for example a ZrO_2_ coating combined with bulk Zr^4+^ doping or a Nb_2_O_5_ coating together with Nb^5+^ bulk substitution.[^25^, ^40^] However, a combination of different compositions as coating and elements as dopants in combination with a core‐shell approach might be needed to benefit from the different positive effects and to overcome the stability issues for NCM materials with Ni contents ≥80 %.

In this work, the combination of Zr^4+^ as a frequently used dopant in commercial Ni‐rich NCM cathode materials with W^6+^‐containing coatings (WO_3_ and Li_2_WO_4_) is thoroughly investigated with a special focus on the impact of processing conditions and post‐processing temperatures. The coating is performed via co‐precipitation of two coating materials (WO_3_ and Li_2_WO_4_) and annealing at the ideal temperatures for the respective targeted coating. Besides comprehensive material characterization to investigate possible crystallographic, morphological, and compositional changes for different processing conditions, the long‐term electrochemical performance in NCM || graphite cells is also evaluated. It is found that the W^6+^‐containing coatings and the Zr^4+^‐based bulk dopant are influenced by each other. The coated samples show an improved electrochemical performance compared to the pristine sample but are inferior to reference samples that were only heat‐treated without any additional coating. This sheds light onto the importance of not only investigating the effect of individual dopants or coatings but also the synergistic effects between both modifications, as well as the effect of a “simple” heat treatment.

## Results and Discussion

### Wet‐coating process

**Figure 1:**
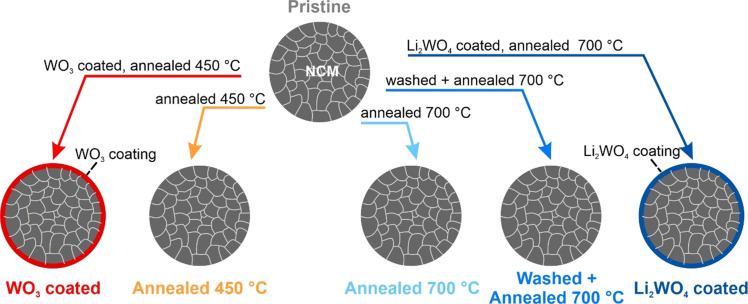
Synthesis conditions and labeling of NCM cathode materials. The scheme shows the different treatments of the pristine material resulting in six samples. The arrows are labeled with the modification procedures, and the resulting samples and their labels are presented below. Further details can be found in Figure 8. Where possible, the color code introduced here is used throughout the manuscript.

The surface modification of Ni‐rich NCM active cathode materials, that is, LiNi_0.83_Co_0.12_Mn_0.05_O_2_ with 0.16 wt % Zr^4+^, was conducted via a simple sol‐gel or co‐precipitation process depending on the applied coating approach. Zr^4+^ and W^6+^ will be used in their most common oxidation states throughout this work although the actual oxidation states were not determined. Annealing temperatures were chosen according to the targeted coating procedure. The resulting samples are schematically presented in Figure 1, introducing the color code that is also used in the following sections. Two types of reference materials were prepared to study the effect of the heat treatment alone. The first reference material underwent the same heat treatment as the coated samples. Those samples are labeled as “annealed” followed by the annealing temperature (450 and 700 °C). For heat treatment at 700 °C in oxygen, an uncoated reference with solvent treatment before annealing was also investigated. This sample underwent the same solvent, drying, and annealing process chain as the coated samples but without a coating and is labeled as “washed+annealed 700 °C”.

### PXRD study of NCM materials

**Figure 2:**
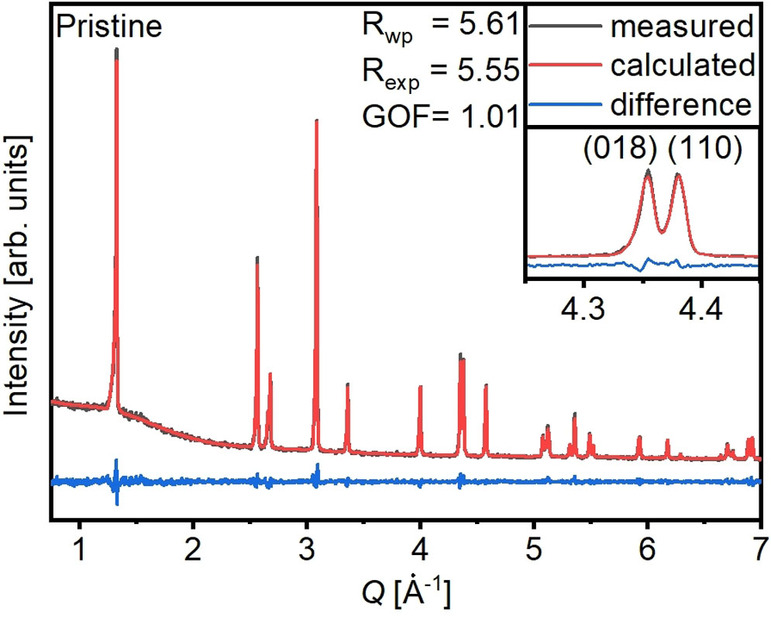
Representative Rietveld refinement of the pristine material showing measured (grey) and calculated (red) data as well as the difference between them (blue). *R*‐weighted pattern (*R*_wp_), *R*‐expected (*R*_exp_), and Godness of fit (GOF) are shown as well. The inset shows the (108)/(110) reflections split up indicating a well‐defined layered structure as presented in Figure S1.

Powder X‐ray diffraction (PXRD) results of the samples are shown in Table 1. An exemplary Rietveld refinement of the pristine sample is presented in Figure 2. No phase impurities were observed, and the refinement as well as the splitting of the (018)/(110) reflections confirm a layered hexagonal α‐NaFeO_2_ structure with a $R\bar{3}m$ space group. Rietveld refinements of the remaining samples and the detailed parameters and results of the Rietveld refinements can be found in Tables S1 and S2 and Figures S2–S6. The PXRD patterns of all surface‐modified cathode materials confirm that all samples have a hexagonal α‐NaFeO_2_ structure with the $R\bar{3}m$ space group. No phase impurities were observed after the different treatments. The lattice parameters and the estimated *c*/*a* ratio only exhibit minor changes. The range of the lattice parameters and the small changes that were observed are in good agreement with literature reports as compared in Table 1.[^37^, ^41^] Slight deviations compared to literature can be explained with the unusual stoichiometry compared to, for example, NCM811. Higher Co contents result in a contraction of the lattice parameters *c* and *a* and an increase in the *c*/*a* ratio, while lower Mn contents result in an increase in the *a* parameter and a decrease of the *c* parameter and the *c*/*a*‐ratio. Both effects are counteracting in the presented samples compared to NCM811.

**Table 1:** Lattice parameters and Li/Ni mixing of investigated NCM materials obtained from Rietveld refinements compared to literature data.

NCM type	Modification	*a* [10^−10^ m]	*c* [10^−10^ m]	*c*/*a*	Li/Ni mixing [%]	Ref.
NCM831205	pristine	2.8675(5)	14.179(4)	4.9445(5)	2.0±0.2	this work
	WO_3_ coated	2.8693(5)	14.189(4)	4.9452(5)	1.6±0.2	
	annealed 450 °C	2.8684(3)	14.184(3)	4.9449(4)	2.1±0.1	
	Li_2_WO_4_ coated	2.8685(4)	14.184(3)	4.9448(4)	1.7±0.2	
	annealed 700 °C	2.8702(3)	14.187(2)	4.9430(5)	2.1±0.1	
	washed+annealed 700 °C	2.8677(3)	14.183(2)	4.9457(2)	2.0±0.1	
NCM811	pristine	2.8713(1)	14.198(1)	4.945	–	[37]
	WO_3_ coated	2.8723(1)	14.198(1)	4.943	–	
NCM811	pristine	2.8708	14.202	4.9471	3	[41]

The degree of cation mixing (Li/Ni mixing) between Li^+^ and Ni^2+^ between their respective slabs (Table 1) only shows differences within the error range and is lower than values reported in literature for NCM811 by Li et al.^[41]^ As the Li/Ni mixing is a measure for a well‐defined hexagonal layered α‐NaFeO_2_ structure in contrast to the rock salt modification (see Figure S1 in the Supporting Information), this indicates that the application of the coatings does not notably affect the crystal structure of the cathode materials.

### Electron microscopy analysis of NCM materials

**Figure 3:**
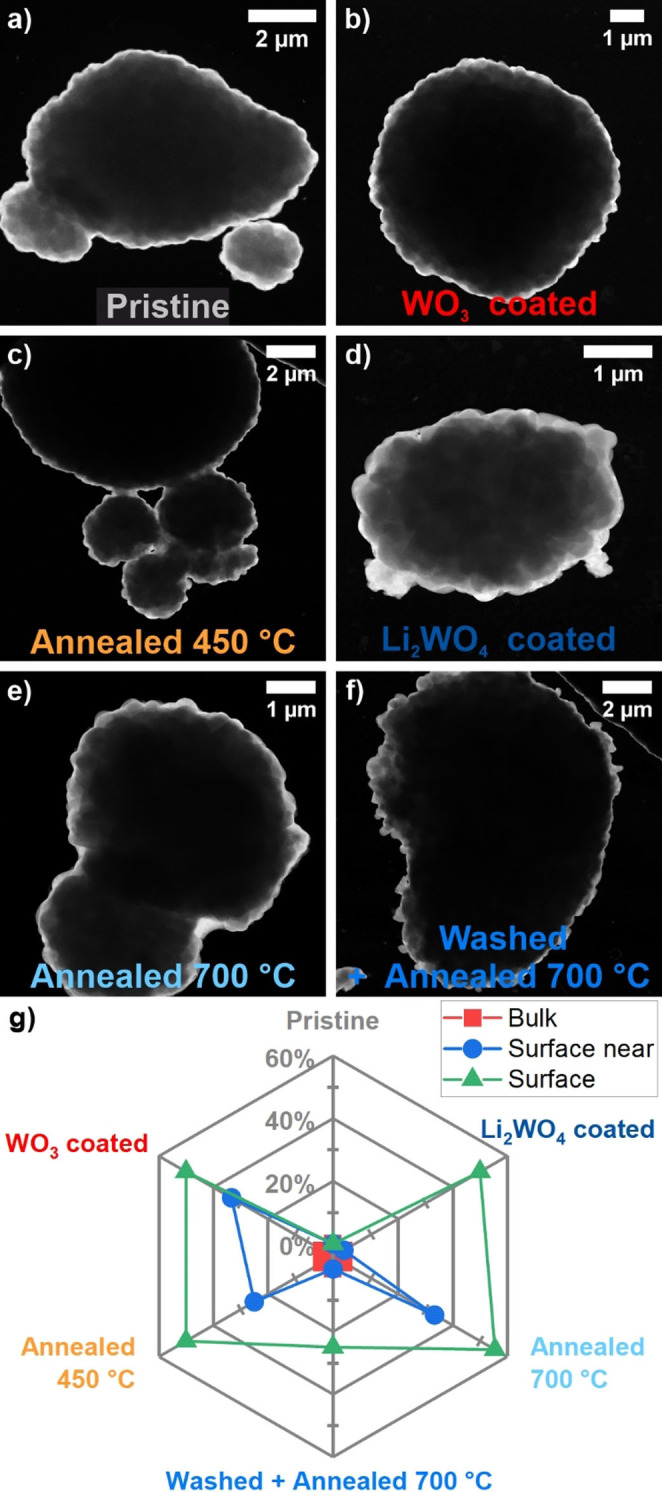
Radar plot visualizing the results of TEM‐EDX analysis shown in Figure S7i. (a–f) Representative TEM images. (g) Percentage of spots measured in bulk, surface near, and surface areas showing traces of Zr^4+^. Representative measurement spots and EDX spectra are shown in Figure S7.

Transmission electron microscopy (TEM) studies combined with energy‐dispersive X‐ray spectroscopy (EDX) measurements were used to analyze the morphology and composition of the pristine and modified cathode materials. TEM analysis was performed on pellets of pressed powders prior to cycling. Representative dark‐field TEM micrographs and corresponding EDX measurements of the samples are shown in Figure S7 and Figure 3. Figure S7a–h and j–m show TEM images of single particles in two magnifications with colored and numbered boxes marking the spots (from 1–5) where the EDX spectra shown in Figure S7i were measured. For the EDX spectra only the regions with major changes between samples (most importantly Zr) are shown. Measurement spots were categorized into bulk (spot 1), surface near (spot 2), and surface measurements (spot 3–5).

The closer investigation of the particle surface via TEM shows significant differences between the samples. While most NCM materials have a smooth and homogenous particle surface, the Li_2_WO_4_ coated sample (Figure 3d and Figure S7g, h) and the washed and annealed sample at 700 °C (Figure 3f and Figure S7l, m) show an uneven surface and the presence of agglomerations. For the washed and annealed at 700 °C sample the agglomerations are small and homogeneously distributed on the whole particle surface. On the other hand, the agglomerations observed on the Li_2_WO_4_ coated particles are larger and clumped together in selected spots. Since other samples, such as the WO_3_ coated one (Figure 3b and Figure S7c, e) and the ones annealed at 450 or 700 °C (Figure 3c, e and Figure S7e, f, j, k) do not show such agglomerations on the particle surfaces, this suggests that the applied treatments strongly influence the particle surface. At 700 °C, the presence of the W^6+^‐based coating seems to promote agglomeration. The discussion of the corresponding EDX results (Figure S7i) focuses on the detection of Zr^4+^ in the respective measurement spots, since it shows the only pronounced intensity differences throughout the samples. Besides that, small amounts of Sr were detected at random spots and W^6+^ can be detected for the coated samples. All measurement spots (11±4) were categorized into bulk, surface near, and surface measurements, and the number of spots with and without Zr^4+^ content were counted. From that, a percentage of spots with Zr^4+^ present could be obtained for each sample and each region of measurement (Figure 3). Interestingly, Zr^4+^ was not detected in the bulk of the particles, while it was found in up to half of the measurements at the particle surface. The absence of Zr^4+^ in the bulk measurements most likely results from its concentration being below the detection limit as it is only present with 0.16 wt %. At the particle surface, Zr^4+^ could be more concentrated or relatively taking up a larger fraction. However, Zr^4+^ was detected neither in the bulk nor on the surface for the pristine NCM material. This suggests that Zr^4+^ is more evenly distributed in the pristine material and might diffuse to the surface to form a coating layer during a heat treatment at temperatures lower than the synthesis temperature.^[42]^ Similar observations have been reported by Yoon et al. for LiNiO_2_, where excess Zr^4+^ doping diffuses to the surface at 650 °C and forms a protective coating layer due to the low bulk solubility limit of Zr^4+^.^[25]^ According to the results of this study, the presence of both W^6+^ and Zr^4+^ at the particle surface appears to lead to mutual influences of both elements. Lower treatment temperatures (450 °C) result in a coating that is less favorable for the electrochemical performance, while the combination of higher temperatures (700 °C) and the use of an aqueous processing route for the coating seems to lead to the formation of agglomerates and, thus, an inferior electrochemical performance as discussed below. Since the resulting surface compounds are a negligible fraction of the sample compared to the bulk, a detailed surface characterization was carried out via X‐ray photoelectron spectroscopy (XPS).

### XPS analysis of NCM materials

**Figure 4:**
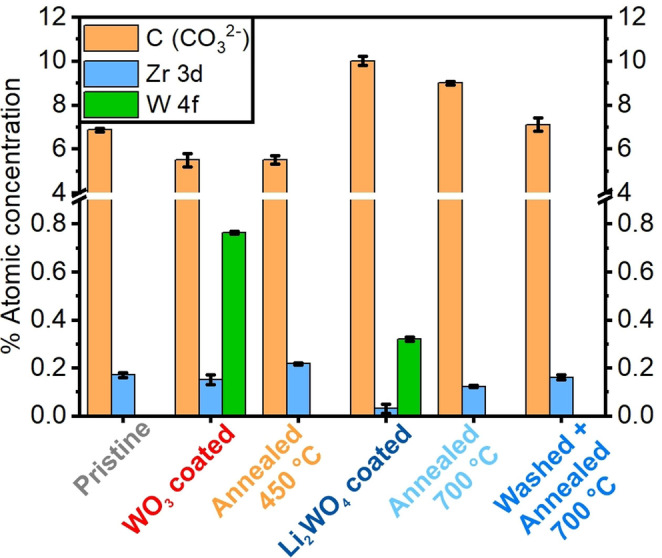
Atomic concentration ratios of the NCM materials derived from the XPS results. Three spectra per element, sample, and position were obtained and the given errors of the % atomic concentrations result from the standard deviation. The sample labels correspond to the ones given in Figure 1.

Surface investigations via XPS analysis were performed; the spectra are shown in Figures S8–S13, and the results are shown in Table S3 and Figure 4. Three spectra per element, position, and sample were measured to ensure reproducibility, and errors given in Figure 4 and Table S3 represent the standard deviation.

For some elements, absolute values in % atomic concentration are given. In other cases, ratios are given for better comparability between samples as transition metal signals might be mitigated by, for example, thicker Li_2_CO_3_ surface layers. From the data in Figure 4, Figures S8–S13, and Table S3, it can be concluded that the relative amount of Li_2_CO_3_ on the particle surface compared to the pristine material is comparable or slightly reduced for the WO_3_ coated sample and the samples annealed at 450 °C. However, it seems to be significantly increased for the samples annealed at 700 °C, washed and annealed at 700 °C, and especially for the Li_2_WO_4_ coated sample. For the latter, a small part of the carbonate Li signal can also result from Li_2_WO_4_ coating because the binding energies for Li are very similar (≈55.1 eV).^[43]^ Since the W^6+^ concentration for this sample is only 0.32 % the majority however most likely results from Li_2_CO_3_.

It is remarkable that the Zr^4+^ content is comparable to the pristine material only for the WO_3_ coated sample and the sample washed and annealed at 700 °C. An increased Zr^4+^ content is detected on the surface of the sample annealed at 450 °C, which could result from Zr^4+^ diffusing to the surface. This is supported by calculations for Zr^4+^ solubility in LiNiO_2_, showing that it is two magnitudes lower at around 400 °C compared to 700–800 °C during the synthesis.^[42]^ On the other hand, the Zr^4+^ content on the surface is slightly reduced for the sample annealed at 700 °C and more significantly reduced for the Li_2_WO_4_ coated sample. One possible explanation is the different surface structure of both samples with the agglomerates seen in the TEM study. Especially for the Li_2_WO_4_ coated sample, it seems likely that the Zr^4+^ is mostly situated in the large agglomerates (≈0.5 μm) on the surface. Large agglomerates however result in less surface containing Zr^4+^ exposed to the XPS beam.

### Electrochemical characterization of NCM materials

The rate capability of the cathode materials was investigated in NCM || Li metal cells to avoid any possible Li metal plating on graphite electrodes and separate the influence of the anode material on overall performance. The C‐rate was therefore only varied upon discharge and kept at 0.2 C during charge to minimize inhomogeneous Li metal plating on the Li‐metal anode happening in organic carbonate electrolytes.^[44]^ The data are shown in Figure S14. Discharge capacities with 4.3 V upper cut‐off voltage at 0.1 C range between 179–193 mAh g^−1^ with the highest discharge capacities for the Li_2_WO_4_ coated sample and the sample annealed at 700 °C. With exception of the sample annealed at 450 °C, all NCM cathode materials show an improved capacity retention behavior compared to the pristine sample and are within the error ranges. In terms of rate capability, the WO_3_ coated sample shows the best performance (Figure S14b), which could result from the good electronic conductivity of WO_3_.^[38]^ Both samples treated at 700 °C and the Li_2_WO_4_ coated sample show intermediate performance ranging between the pristine material and the WO_3_ coated sample in terms of rate capability. Discharge capacities of 158–163 mAh g^−1^ are reached at a discharge rate of 3 C for all samples except for the pristine and the one annealed at 450 °C.

Long‐term charge/discharge cycling experiments were performed in NCM || graphite cells until 80 % state‐of‐health (SOH) was reached (SOH with reference to the 1st discharge capacity at a rate of 0.33 C, corresponding to cycle No. 5). Four formation cycles were conducted at 0.1 C (=19 mA g^−1^) to allow reliable comparison between datasets,^[45]^ while the following long‐term cycling took place at 0.33 C with two recovery cycles at 0.1 C every 100 cycles. Table S4 shows the initial coulombic efficiencies (CEs), initial discharge capacities at 0.1 and 0.3 C after formation, and the cycle where the pre‐defined end of life has been reached for all prepared samples.

**Figure 5:**
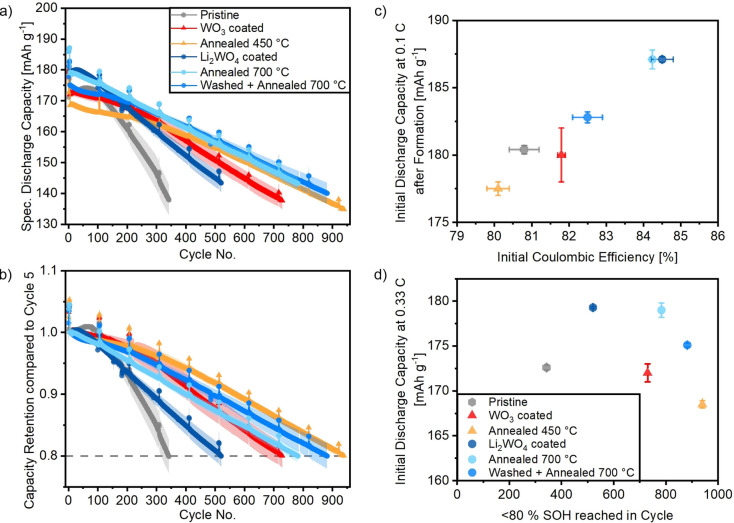
Electrochemical cycling data of NCM || graphite full cells between 2.8–4.2 V. The first four cycles were conducted at 0.1 C, while the following long‐term cycling took place at 0.33 C with two cycles at 0.1 C each 100th cycle. (a) Specific discharge capacities and (b) capacity retentions compared to the first cycle at 0.33 C. Error bars: standard deviation of three cells for each sample. (c) Initial discharge capacity at 0.1 C after 3 formation cycles vs. initial CE. (d) Initial discharge capacity at 0.33 C after formation vs. end‐of‐life cycle. The CE for the pristine material is 99.7 % during cycling, while it is above 99.9 % for all other samples.

The electrochemical performance for all samples is compared in Figure 5. The pristine NCM || graphite cell displays an initial discharge capacity of 180 mAh g^−1^ at 0.1 C and 173 mAh g^−1^ at 0.33 C in a cell voltage window of 2.8–4.2 V, while the end of life is already reached after approximately 343 cycles with an average CE of 99.8 %. The WO_3_ coated sample shows the same initial discharge capacity but reaches the end of life at approximately 730 cycles. The corresponding reference annealed at 450 °C has a slightly lower initial capacity but shows the most stable cycling performance with around 940 cycles. All samples that were annealed at 700 °C show higher initial capacities of 183–187 mAh g^−1^ at 0.1 C. Despite being more stable than the pristine material, the Li_2_WO_4_ coated sample already reaches the end of life after around 521 cycles. Again, in comparison the heat‐ and solvent‐/heat‐treated samples show an improved cycling stability with the end of life reached after approximately 783 cycles (annealed at 700 °C) or even approximately 882 cycles (washed+annealed at 700 °C). All treated samples exhibit a CE above 99.9 %. In addition, it is remarkable that all three reference samples, although, for example, heat‐treated at different temperatures and in different atmospheres, show a very similar cycling behavior from the 500th cycle onwards. As the pristine Ni‐rich cathode material and especially the experimental conditions (including electrode mass loading, cell setup, cycling procedure, etc.) are not standardized throughout literature it is difficult to compare the obtained values to previously reported ones. However, the cycle life of 800 to almost 1000 cycles obtained with lab‐scale LIB full‐cells with a Ni‐rich cathode (containing 83 % Ni) appears superior compared to literature reports, which range from around 200 to 900 cycles.[^37^, ^38^, ^46^, ^47^] The remaining capacity fading can likely be attributed to bulk instabilities arising from the high Ni content and hence micro‐crack formation and contact loss for parts of the cathode material. A comparison with the results from the sections above in relation to the electrochemical performance will be further discussed below.

### Post‐mortem analysis: comparison of pristine and cycled materials via SEM

**Figure 6:**
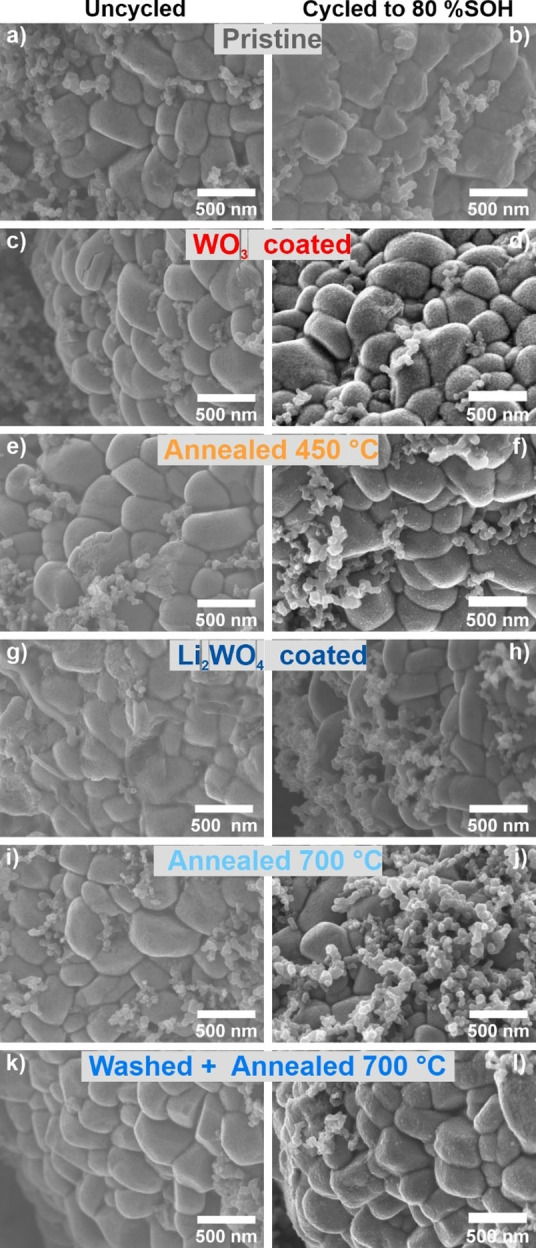
SEM images of various samples before (left) and after (right) cycling until 80 % SOH was reached. The images were recorded with a magnification of 50 k. SEM images with lower magnifications are shown in Figure S15.

Post‐mortem analysis of the cells after reaching the end of life was conducted ex situ by opening the cells in an Ar‐filled glovebox. Scanning electron microscopy (SEM) and EDX analysis of electrodes was conducted before and after cycling. The results at lower magnifications are displayed in Figure S15 and do not show any significant differences. Images at higher magnifications are compared in Figure 6 and show significant differences for selected materials. Before cycling most particle surfaces have a quite clean appearance. Only the pristine material and the Li_2_WO_4_ coated sample show residues on the surface to very different extents. The pristine sample only shows negligible amounts, while the Li_2_WO_4_ coated sample is covered by multiple large parts of residues. However, this changes significantly after cycling to 80 % SOH, where the surface of the Li_2_WO_4_ coated sample is cleaned and looks as fresh as all the other materials before and after cycling. For the pristine sample, however, the opposite is the case, and the surface is covered by a continuous layer of residues [most likely cathode electrolyte interphase (CEI) or electrolyte decomposition products] even though it was only cycled for 343 cycles. This new surface layer might also be the reason for the poor cycling performance of the pristine material shown in Figure 5. For the Li_2_WO_4_ coated sample, on the other hand, the decomposition products of the surface residues before cycling might lead to an accelerated capacity fading.

## Discussion

**Figure 7:**
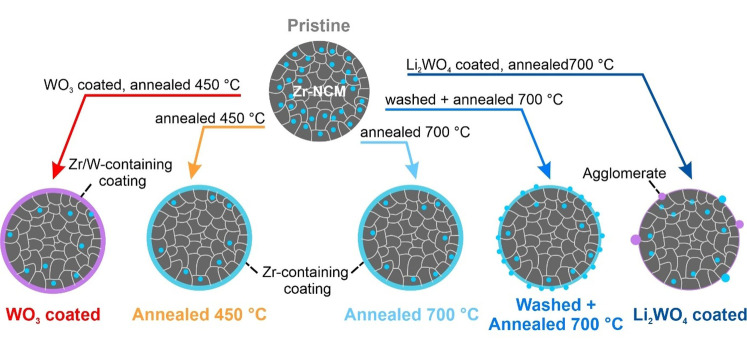
Synthesis conditions and schematic representation of the sample surfaces. The scheme shows the different treatments of the pristine material resulting in six samples. The arrows are labeled with the modification procedures and the resulting sample and label are presented below. Light blue dots in the particle indicate Zr^4+^ doping, while light blue dots on the surface indicate Zr^4+^ containing agglomerates. Light blue layers indicate Zr^4+^ containing coatings, while purple layers indicate Zr^4+^ and W^6+^ containing coatings.

As indicated above, no significant differences in terms of the crystallographic structure were observed via PXRD. In the observed range of lattice parameters and cation mixing those parameters do not have a major impact on the electrochemical performance. More pronounced differences can be seen via TEM investigations as schematically shown in Figure 7. It seems likely that the formation of large agglomerates observed for the Li_2_WO_4_ coated sample are linked to the material's inferior long‐term cycling. However, smoother particle surfaces do not necessarily correspond to an improved cycling stability. While this is true for the WO_3_ coated sample and the samples annealed at 450 and 700 °C, it is not true for the pristine material and the sample washed and annealed at 700 °C. While the pristine material shows a smooth particle surface but experiences fast degradation during electrochemical cycling, the smaller agglomerates on the surface of the washed and annealed at 700 °C sample do not seem to have a negative influence. The TEM‐EDX results suggest that the Zr content on the surface is increasing during annealing/treatment steps. This is more pronounced at lower temperatures due to lower solubility limits for Zr. According to calculations this process should be reversible, and the final state might be tuned by heating time and temperature.^[42]^ The SEM results suggest that the faster electrochemical degradation can be linked to either the removal of surface residues (Li_2_CO_3_) or the generation of significant amounts of new surface residues. In both cases decomposition products of the residues likely act as “enabler” for parasitic side reactions. Samples with visibly “clean” particle surfaces generally showed better cycling performance. For all treated samples, a correlation between XPS results and electrochemical performance can be seen. A better electrochemical performance is commonly observed for samples with lower amounts of surface Li_2_CO_3_ (lower Li 1s/Ni 3p ratio) and a higher Zr^4+^ concentration in the surface or Zr 3d/Ni 3p ratio. However, since this is not the only relevant parameter, it is only a qualitative observation.

To summarize the findings above, multiple conclusions can be drawn: a main one is that it is generally very important in coating studies to also include heat‐ and solvent‐treated reference materials to study the impact of the involved processes on the resulting electrochemical performance. This could avoid misinterpretations, such as overestimations of the impact of coatings, where the observed improvements are at least partially due to the treatment conditions and not due to the chemistry of the coating. In the specific case shown in this work, the treatment of the Zr^4+^ present in the sample seems to have a more beneficial effect than applying an additional W^6+^ containing coating. This probably results from the synergistic effects of both elements depending on the solvents and the annealing temperatures and atmospheres that were used. Even though more Zr^4+^ diffuses to the particle surface at 450 °C, this treatment still seems to be more beneficial in combination with a W^6+^‐containing coating carried out in isopropanol. Higher annealing temperatures and the use of water as a processing solvent during the coating process, however, lead to the formation of large agglomerations with Zr^4+^ and W^6+^ compounds instead of a uniform Li_2_WO_4_ coating. This sample still has a better performance than the pristine material but also a significantly worsened performance than the heat‐ and solvent‐treated references.

## Conclusions

This work provides highly relevant insights in combining different elements as surface coating and bulk doping in nickel‐cobalt‐manganese (NCM) cathode materials. Significant influences between both modification approaches could be shown depending on the solvent used for the sol‐gel synthesis as well as the type of annealing gas and the applied temperature in the subsequent treatment steps. A broad range of surface textures from smooth over smaller to larger agglomerates could be observed, clearly showing that combining doping and coating is not straightforward in the shown cases.

On the route towards low‐cost and more sustainable cathode materials with increasing Ni contents >80 % for high‐energy lithium‐ion battery cells, however, combining both approaches can be of utmost positive impact. It is therefore crucial to investigate the mutual influences whenever coating and doping are combined. Therefore, either beneficial synergistic effects of doping and coating are necessary or a dopant with low tendency towards segregation needs to be incorporated in the bulk, while the coating remains on the surface to prevent mutual influences. Last but not least, this work highlights the importance of including heat‐ and/or solvent‐treated reference samples together with the, for example, coated material. Only this approach can unequivocally reveal the beneficial effect of the applied coating.

## Experimental Section

### Material preparation

The used Ni‐rich cathode active material was LiNi_0.83_Co_0.12_Mn_0.05_O_2_ (NCM831205; “S85EL‐1^st^ sintering”, Ronbay Technology, China) with 0.16 wt % Zr^4+^ [determined by inductively coupled plasma optical emission spectrometry (ICP‐OES)]. As coating precursors, ammonium tungstate [(NH_4_)_10_H_2_(W_2_O_7_)_6_, 99.99 %, Sigma‐Aldrich] and lithium tungstate (Li_2_WO_4_, 99 %, Alfa Aesar) were used for WO_3_ and Li_2_WO_4_ coatings, respectively. PXRD was measured to verify the purity of the pristine precursor material, revealing that Li_2_WO_4_ was partially hydrated to [H_2_O]_4_Li_14_[WO_4_]_7_. Since the synthesis was performed either in water or isopropanol (not dried), those trace amounts of water were just taken into account for the coating/active material ratio.

**Figure 8:**
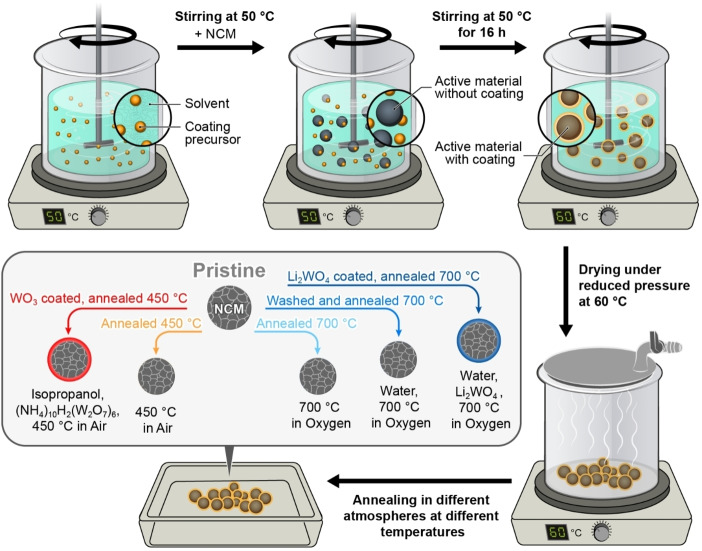
Synthesis procedure and labeling of the samples. The schematic illustration shows the coating process of the precursors (1 wt % of the coating material) via a dispersion in the respective solvent and subsequent addition of the active material (Ni‐rich NCM). The resulting dispersion has a solid content of around 40 %. After moderate stirring at 50 °C for 16 h, the solvent was evaporated at 60 °C under reduced pressure. The coated active materials were then annealed at the desired temperature in different atmospheres.

A schematic representation of the synthesis conditions can be found in Figure 8. The coating was performed with the precursor (stoichiometric amounts to achieve a coating of 1 wt %) being dissolved in the respective solvent (deionized H_2_O for Li_2_WO_4_ or isopropanol for WO_3_). This mixture was stirred with a magnetic stirrer at 50 °C for approximately 1 h to achieve a homogeneous dispersion before the active material was added (solid content: ≈40 %). After continuous stirring at 50 °C for 16 h, the solvent was evaporated at 60 °C under reduced pressure with a Büchi rotary evaporator. The obtained powder was then annealed in the respective atmospheres using tube or muffle furnaces (Nabertherm) at different temperatures (WO_3_: 450 °C in air; Li_2_WO_4_: 700 °C in O_2_). The final material was hand‐grinded with mortar and pestle for homogenization before further processing. The presence of tungsten was confirmed via EDX. To study the effect of the heat treatment alone, two types of reference samples were prepared. The first reference sample underwent the same heat treatment as the coated samples (abbreviated as “Annealed 450 °C” or “Annealed 700 °C”). For the heat treatment at 700 °C in oxygen also a reference dispersed in water was investigated (abbreviated as “Washed+Annealed 700 °C”). This sample underwent the same solvent, drying under reduced pressure and annealing process as the Li_2_WO_4_ coated samples but without the coating.

### Material characterization

The chemical composition of the pristine Ni‐rich cathode material was determined using ICP‐OES (Spectro ARCOS EOP) with an axial positioned plasma torch. Measurement conditions were applied according to Vortmann‐Westhoven et al.^[48]^ and Evertz et al.^[49]^

Powder samples for XRD were prepared on polyvinylacetate‐foil. Diffractograms were obtained using a STOE Stadi P, equipped with a Mythen 1k detector using MoK_α1_ radiation. The samples were measured in transmission in 0.015° steps (continuous scan, 150 s per °) covering a 2*θ* range from 1.5 to 47°. Rietveld refinements^[50]^ were performed with Topas Academic V6 (Bruker AXS GmbH) based on a hexagonal α‐NaFeO_2_ structure with space group $R\bar{3}m$ using the fundamental parameters approach.^[51]^ For the refinements, Li was assumed to occupy 3*a* sites, while Ni, Co, and Mn were assumed to occupy 3*b* sites. Additional constraints were used to refine Ni moving from the 3*b* to the 3*a* site and at the same time Li from the 3a to the 3b site to account for Li/Ni mixing.

The particle morphology and elemental distribution of pristine cathode materials were investigated by SEM and EDX using a Carl Zeiss AURIGA field emission microscope with a Schottky field emitter as electron source. The typical accelerating voltage was 3 kV. EDX was determined via a X‐Max 80 mm^2^ detector (Oxford Instruments) at 20 kV operating voltage. Cycled NCM || graphite cells were disassembled in the discharged state at 2.8 V in an argon‐filled glovebox and the cathode surfaces were rinsed with 200 μL of ethyl‐methyl carbonate (EMC, BASF) to remove salt impurities prior to analysis. After a short drying period under reduced pressure, the electrodes were transferred into the SEM via a vacuum‐sealed sample holder to avoid exposure to atmospheric air.

Powdered samples were prepared by placing one drop (10 μL) of a diluted active material solution in water (0.1 mg mL^−1^) on a carbon‐coated copper grid and by letting it dry at room temperature for scanning transmission electron microscopy (STEM) and EDX. STEM and EDX measurements were carried out with a FEI TECNAI F30 S‐TWIN transmission electron microscope equipped with a field emission gun and working at 300 kV. STEM images were collected by a FISCHIONE high‐angular annular dark field (HAADF) detector. EDX spectra were acquired at a tilt angle of 20° using an EDAX EDAMIII detector and Emispec ES Vision software.

XPS measurements were conducted on an Axis Ultra DLD (Kratos) at 10–8 mbar with monochromatic AlK_α_ X‐rays (*hν*=1486 eV, 10 mA emission current, and 12 kV acceleration voltage). A charge neutralizer was used to compensate for charging of the samples. For core level spectra, a pass energy of 40 eV, a step interval of 0.2 eV, and an emission angle of 0° to the sample normal were chosen. Recorded core spectra were fitted with CASA XPS V2.3.22PR1.0. The binding energy (B.E.) scale was referenced to the C 1s C−H/C−C peak (B.E.=284.5 eV). Three different spots per sample were measured to ensure a high reproducibility.

### Electrode preparation

The Ni‐rich positive electrodes consisted of 94 wt % active material, 3 wt % carbon black as conductive agent (Super C65, Imerys Graphite & Carbon), and 3 wt % poly(vinylidene difluoride) (PVdF) as binder (Solef 5130, Solvay). *N*‐methyl‐2‐pyrrolidone (NMP, anhydrous, purity: 99.5 %, Sigma‐Aldrich) was used as solvent, reaching a solid content of around 50 wt %. For electrode paste preparation, the PVdF binder, Super C65, and active material were mixed in dry state followed by the addition of NMP. The electrode paste was then homogenized by a high‐energy disperser (Dissolver Dispermat LC30, VMAGetzmann GmbH) at a speed of 15000 rpm for 1 h. After complete dispersion, the paste was coated on Al foil (20 μm, Nippon foil, previously washed with ethanol) using a doctor‐blade (Zehntner GmbH) and an automatic film applicator (Sheen Instruments). Afterwards, the electrode sheets were dried in an atmospheric oven at 80 °C for 2 h, calendared with a gap of 5 or 20 μm, respectively, to reach a porosity of around 35 %, punched out in 14 mm diameter disks, and finally dried in a Büchi B‐585 glass drying oven under reduced pressure (<5×10^−2^ bar) at 120 °C for 12 h. The average cathode active material mass loadings and areal capacities were (i) around 5.0±0.2 mg cm^−2^ (0.96±0.3 mAh cm^−2^) for investigations in NCM || Li metal cells and (ii) 12.0±0.5 mg cm^−2^ (2.3±0.5 mAh cm^−2^) for NCM || graphite full‐cell investigations. The areal capacities are based on the 2nd cycle discharge capacity from NCM || Li metal cells (19 mA g^−1^, 2.9–4.3 V).

The negative electrodes used for NCM || graphite full‐cell investigations consisted of 95 wt % commercial synthetic graphite as the active material, 1.5 wt % styrene‐butadiene‐rubber (SBR, SB5521, LIPATON, Polymer Latex GmbH), and 3.0 wt % sodium‐carboxymethyl cellulose (Na‐CMC, Walocel CRT 2000 PPA12, Dow Wolff Cellulosics) as binders, and 0.5 wt % carbon black as conductive agent (Super C65, Imerys Graphite & Carbon). Deionized water was used as solvent for paste preparation. The paste viscosity was optimized to reach a solid content of around 40 wt % and homogenized as described above. The negative electrode paste was cast onto copper foil (10 μm, Nippon foil). After drying and calendaring the graphite sheets to achieve 30 % porosity, Ø=15 mm circular electrodes were punched out, and the electrodes were dried in a Büchi B‐585 glass drying oven under reduced pressure (<5×10^−2^ bar) at 120 °C for 12 h. The average active mass loading of the negative electrodes was around 7±1 mg cm^−2^, resulting in an areal capacity of around 2.5±0.3 mAh cm^−2^ based on the practical capacity of graphite (≈350 mAh g^−1^) obtained from the 2nd cycle discharge capacity from graphite || Li metal cells.

**Cell assembly and electrochemical characterization**: Electrochemical investigations were carried out in two‐electrode configuration in coin cells (CR2032, Hohsen Corporation). All cells were assembled in dry room atmosphere with a dew point of at least −50 °C (relative humidity of 0.16 %). 1 m LiPF_6_ in 3 : 7 vol % EC/EMC (Solvionic) with 2 wt % vinylene carbonate (VC, 99.9 %) as solid electrolyte interphase (SEI) additive was used as electrolyte. A polymer membrane (1‐layer, 16 mm Ø, Celgard 2500, Celgard) soaked with 35 μL of electrolyte was used as separator. The C‐rate capability and long‐term cycling stability of Ni‐rich cathode materials were investigated in NCM || Li metal cells and NCM || graphite full‐cells, respectively. At least three cells per sample were assembled to ensure a high reproducibility of our results. The standard deviation between cells is represented as error bars in the corresponding figures.

For NCM || Li metal cell investigations, Ni‐rich layered oxides as positive electrode (Ø=14 mm; 0.96±0.3 mAh cm^−2^) and a Li metal negative electrode [Ø=15 mm, lithium metal foil, 500 μm; battery grade: purity ≥99.9 %, China Energy Lithium (CEL Co.)] were used. For NCM || graphite full‐cell investigations, Ni‐rich layered oxides as positive electrode (Ø=14 mm; 2.3±0.5 mAh cm^−2^) and graphite as negative electrode (Ø=15 mm) were considered. The negative/ positive (N/P) capacity balancing ratio was set to 1.15 based on the 2nd cycle discharge capacity from Li metal cell investigations.

Electrochemical properties were investigated via constant‐current (CC) charge‐discharge cycling on a Maccor Series 4000 battery tester (Maccor, Inc.) at 20 °C. The specific current for a rate of 1 C was defined as 190 mA g^−1^. The rate capability of cathode materials at different upper cut‐off cell voltages was investigated in NCM || Li metal cells according to the following procedure: 6 h at open‐circuit‐voltage (OCV) followed by two formation cycles at 0.1 C, three cycles at 0.2 C, and five cycles at 0.33, 0.5, 1, and 3 C each. For discharges rate above 0.2 C, asymmetric tests were performed, and the charge rate was kept to 0.2 C. The cell voltage window up to this point was 2.9–4.3 V. After the C‐rate investigations, cells were cycled at 0.1 C for two cycles, followed by 15 cycles at 0.33 C at different upper cut‐off cell voltages: 4.3, 4.4, and 4.5 V.

The long‐term stability of cathode materials was evaluated in NCM || graphite full‐cells within the cell voltage range of 2.8–4.2 V. For that, these cells were cycled for four cycles at 0.1 C for interphase formation, followed by cycling at 0.33 C until dropping to 80 % SOH. Each 100th cycle, cells were cycled at 0.1 C again for two cycles to evaluate the capacity retention. After each charge step, a constant‐voltage (CV) step was performed with the limiting conditions of either achieving a time limit of maximum 30 min or when the specific current reached values ≤0.05 C.

## Conflict of interest

The authors declare no conflict of interest.

## Supporting information

As a service to our authors and readers, this journal provides supporting information supplied by the authors. Such materials are peer reviewed and may be re‐organized for online delivery, but are not copy‐edited or typeset. Technical support issues arising from supporting information (other than missing files) should be addressed to the authors.

Supporting Information
