# Supplementary material for: Synergistic Effects of Surface Coating and Bulk Doping in Ni‐Rich Lithium Nickel Cobalt Manganese Oxide Cathode Materials for High‐Energy Lithium Ion Batteries
Source: ChemSusChem. 2021 Dec 2;15(4):e202102220. doi: 10.1002/cssc.202102220 (PMC9300204; doi:10.1002/cssc.202102220)
Supplement: Supplementary file 1 — Supporting Information [file CSSC-15-0-s001.pdf]

ChemSusChem

Supporting Information

Synergistic Effects of Surface Coating and Bulk Doping in Ni-Rich Lithium Nickel Cobalt Manganese Oxide Cathode Materials for High-Energy Lithium Ion Batteries

Friederike Reissig, Martin Alexander Lange, Lukas Haneke, Tobias Placke, Wolfgang G. Zeier, Martin Winter, Richard Schmuch,* and Aurora Gomez-Martin*© 2021 The Authors. ChemSusChem published by Wiley-VCH GmbH. This is an open access article under the terms of the Creative Commons Attribution License, which permits use, distribution and reproduction in any medium, provided the original work is properly cited.

Comparison of Rock-Salt and Layered Hexagonal Structure

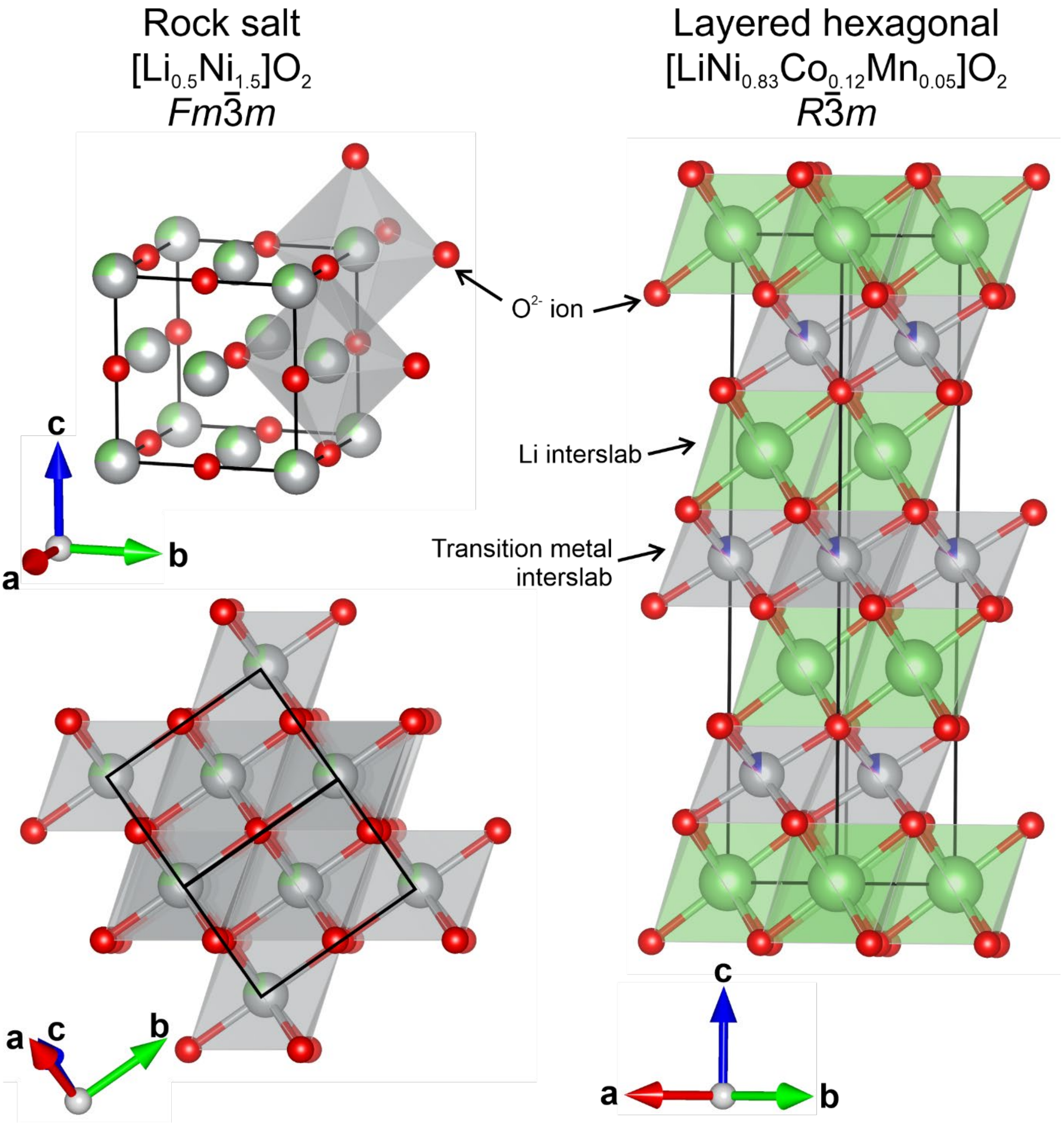

Figure S1. Comparison of different structures for lithium nickel oxide ($LiNiO_2$, LNO). Left rock salt structure of Li^+ poor LNO, *e.g.,* $Li_{0.5}Ni_{1.5}O_2$ in two different orientations showing the common orientation (upper) and a comparison to the layered hexagonal structure (lower). Right: Layered hexagonal structure of the pristine material $LiNi_{0.83}Co_{0.12}Mn_{0.05}O_2$. Red: Oxygen, Grey: Nickel, Green: Lithium, Blue: Cobalt, Purple: Manganese.

X-Ray Diffraction Study of NCM Materials

Table S1: Information on the structure and parameters used for the refinement as well as introduction of the refined parameters *z*(O), Li_{mix}, beq(O) and beq(TM).

Atom	Wyckoff Site	*x* or *a*	*y* or *b*	*z* or *c*	Occupancy	Isotropic thermal factor beq
**O**	*6c*	0	0	z (O)	1	beq(O)
**Li1**	*3a*	0	0	0	0.961 – Li_{mix}	2
**Ni2**	*3a*	0	0	0	Li_{mix}	2
**Ni1**	*3b*	0	0	0.5	0.829 - Li_{mix}	beq(TM)
**Co1**	*3b*	0	0	0.5	0.122	beq(TM)
**Mn1**	*3b*	0	0	0.5	0.049	beq(TM)
**Li2**	*3b*	0	0	0.5	Li_{mix}	beq(TM)

Table S2: Results of the Rietveld refinements: R-weighted pattern R_{wp}, R expected R_{exp}, Goodness of fit GOF, lattice parameter *a* and *c*, unit cell volume *V* Li/Ni mixing Li_{mix}, *z*-Position and isotropic thermal factors.

Modification	Pristine	WO_3 coated	Annealed 450 °C	Li_2WO_4 coated	Annealed 700 °C	Washed + Annealed 700 °C
**R_{wp}**	5.61	5.53	6.28	5.52	6.40	7.07
**R_{exp}**	5.55	5.52	5.74	5.52	5.61	5.36
**GOF**	1.01	1.00	1.09	1.00	1.14	1.32
a **[10^{-10}m]**	2.8675(5)	2.8693(5)	2.8684(3)	2.8685(4)	2.8702(3)	2.8677(3)
c **[10^{-10}m]**	14.179(4)	14.189(4)	14.184(3)	14.184(3)	14.187(2)	14.183(2)
V **[10^{-30}m^3]**	100.97(4)	101.17(4)	101.07(3)	101.07(4)	101.22(3)	101.01(3)
c/a	4.9445(5)	4.9452(5)	4.9449(4)	4.9448(4)	4.9430(5)	4.9457(2)
**Li_{mix}**	0.02(2)	0.01(2)	0.02(1)	0.01(9)	0.02(1)	0.02(1)
**Li/Ni mixing [%]**	2.0 ± 0.2	1.6 ± 0.2	2.1 ± 0.1	1.7 ± 0.2	2.1 ± 0.1	2.0 ± 0.1
z **(O)**	0.241(2)	0.241(2)	0.241(2)	0.241(2)	0.241(2)	0.241(1)
**beq(O)**	0.6(6)	0.5(6)	0.6(4)	0.8(6)	0.6(4)	0.6(4)
**beq(TM)**	0.3(2)	0.4(2)	0.3(2)	0.5(2)	0.2(2)	0.3(2)

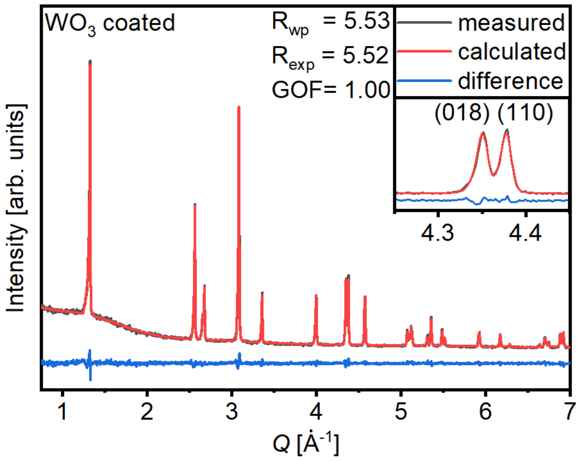

Figure S2: Rietveld refinement of the WO_3 coated NCM material showing measured (grey) and calculated (red) data as well as the difference between them (blue). R_{wp}, R_{exp} and GOF are shown as well. The inlayer shows the (108)/(110) reflections split up indicating a well-defined layered structure as presented in Figure S1.

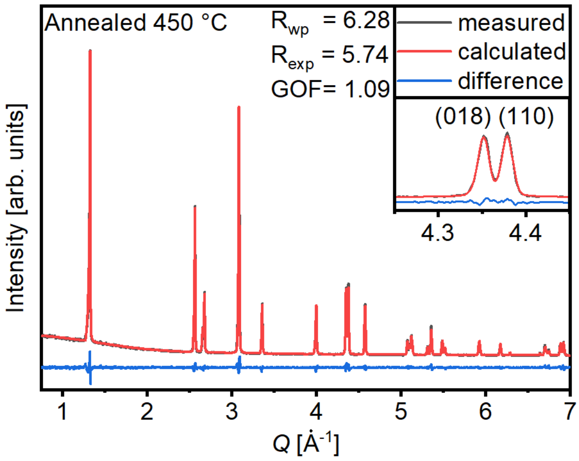

Figure S3: Rietveld refinement of the NCM material annealed at 450 °C showing measured (grey) and calculated (red) data as well as the difference between them (blue). R_{wp}, R_{exp} and GOF are shown as well. The inlayer shows the (108)/(110) reflections split up indicating a well-defined layered structure as presented in Figure S1.

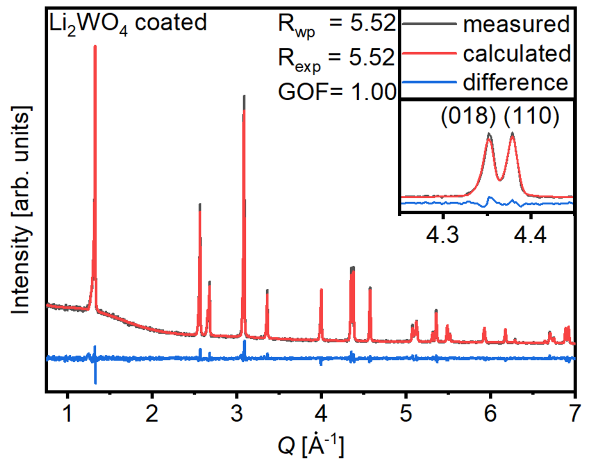

Figure S4: Rietveld refinement of the Li_2WO_4 coated NCM material showing measured (grey) and calculated (red) data as well as the difference between them (blue). R_{wp}, R_{exp} and GOF are shown as well. The inlayer shows the (108)/(110) reflections split up indicating a well-defined layered structure as presented in Figure S1.

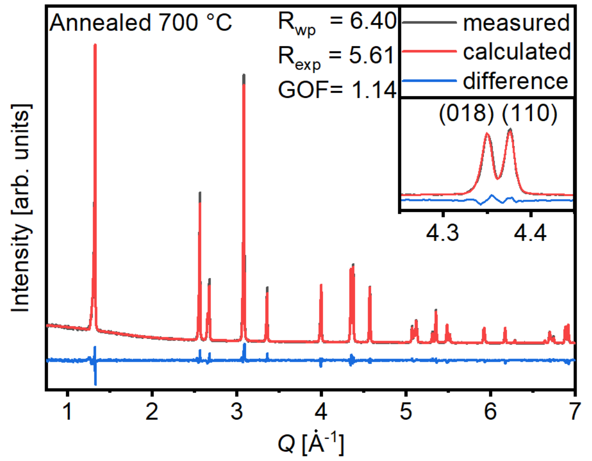

Figure S5: Rietveld refinement of the NCM material annealed at 700 °C showing measured (grey) and calculated (red) data as well as the difference between them (blue). R_{wp}, R_{exp} and GOF are shown as well. The inlayer shows the (108)/(110) reflections split up indicating a well-defined layered structure as presented in Figure S1.

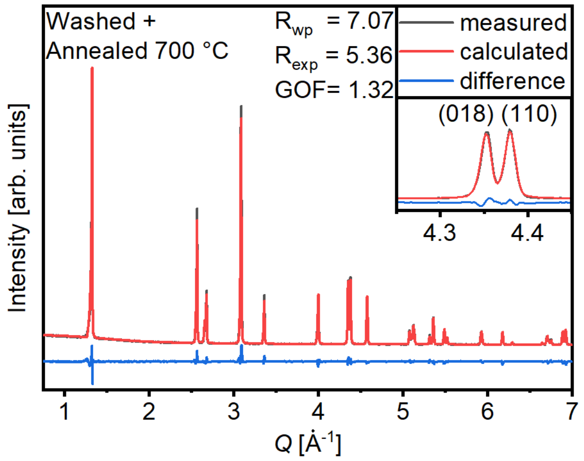

Figure S6: Rietveld refinement of the NCM material washed and annealed at 700 °C showing measured (grey) and calculated (red) data as well as the difference between them (blue). R_{wp}, R_{exp} and GOF are shown as well. The inlayer shows the (108)/(110) reflections split up indicating a well-defined layered structure as presented in Figure S1.

TEM Analysis of NCM Materials

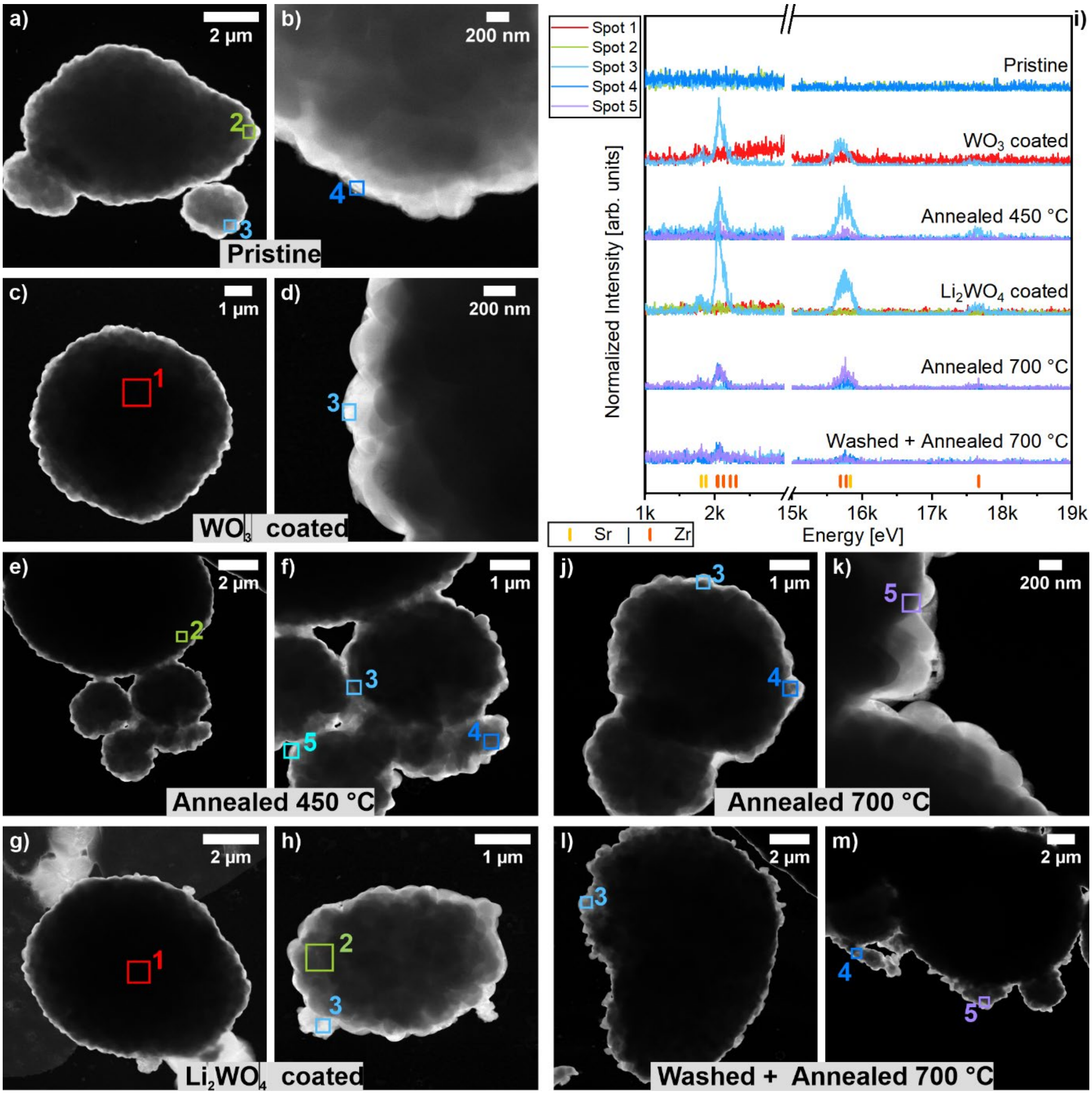

Figure S7: Dark-field TEM images and TEM EDX results. a) - h) and j) - m) TEM images of the secondary particles of the active material. Color coded and numbered are the spots where EDX was measured **i)** EDX spectra corresponding to the spots marked in the TEM images with the same color code. Energy regions of Sr and Zr are shown and the peak positions are marked in yellow and orange, respectively.

XPS analysis

Table S3. XPS analysis of NCM cathode materials. The values were obtained from fitting the core spectra with CASA XPS V2.3.22PR1.0. Reference binding energies were taken from the National Institute of Standards and Technology (NIST) XPS Database[43]. For some elements absolute values in % atomic concentration are given. In other cases, ratios are given for better comparability. The sample labels correspond to the ones given in Figure 1.

	**Pristine**	**WO_3 coated**	**Annealed 450 °C**	**Li_2WO_4 coated**	**Annealed 700 °C**	**Washed + Annealed 700 °C**
**Zr 3d**	0.17 ± 0.01	0.15 ± 0.02	0.217 ± 0.005	0.03 ± 0.02	0.123 ± 0.005	0.16 ± 0.01
**Zr 3d / Ni 3p**	0.040 ± 0.004	0.032 ± 0.004	0.039 ± 0.001	0.02 ± 0.01	0.039 ± 0.003	0.039 ± 0.004
**C (CO_3^{2-})**	6.86 ± 0.09	5.5 ± 0.3	5.5 ± 0.2	10 ± 0.2	8.99 ± 0.08	7.1 ± 0.3
**Li(CO_3^{2-}) / Li(NCM)**	4 ± 2	2.3 ± 0.8	2.9 ± 0.8	13 ± 7	5.8 ± 0.4	2.1 ± 0.5
**Li 1s / Ni 3p**	3.7 ± 0.1	3.0 ± 0.1	2.4 ± 0.1	8.0 ± 0.4	5.5 ± 0.4	3.7 ± 0.2
**W 4f**	--	0.763 ± 0.005	--	0.320 ± 0.008	--	--

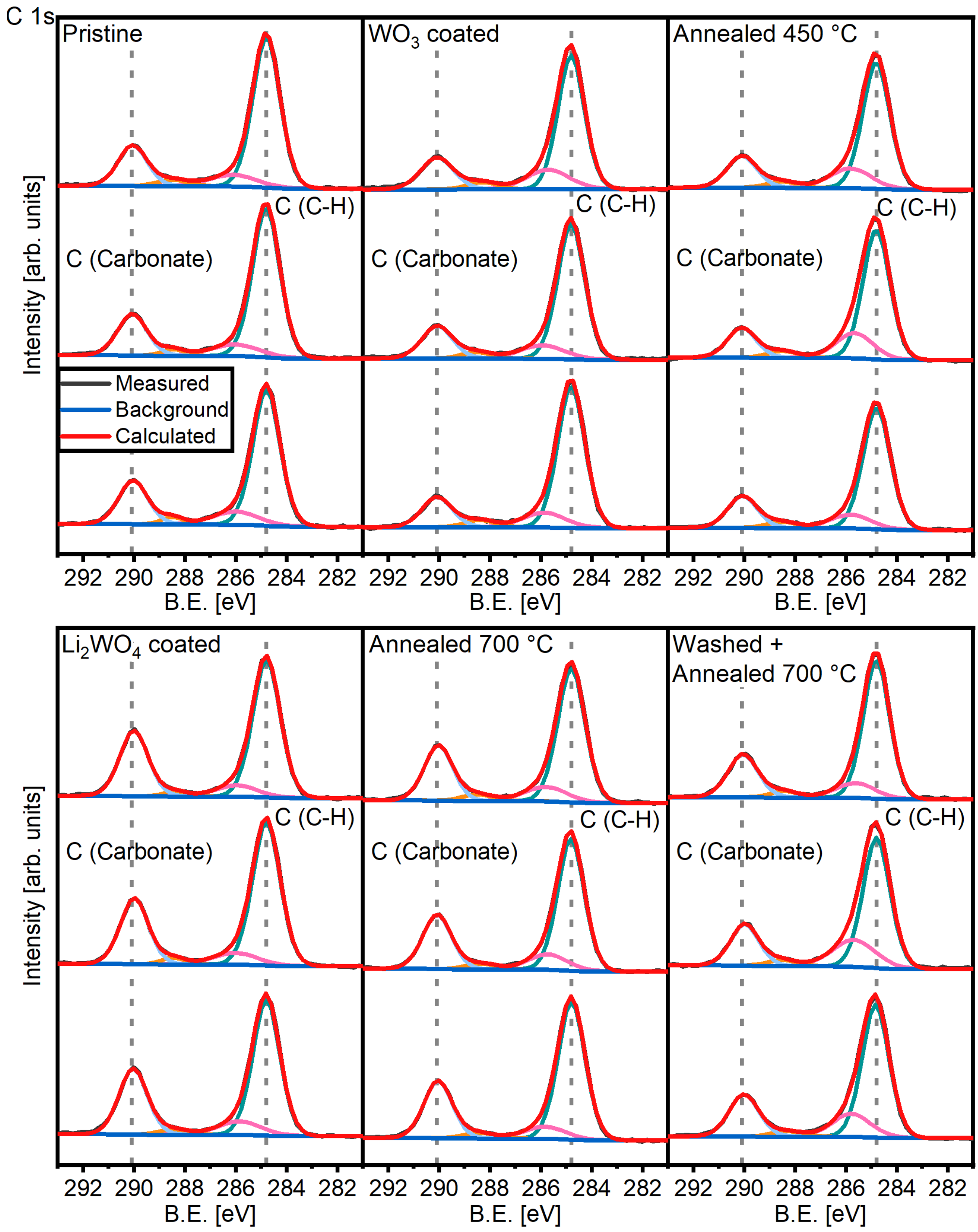

Figure S8*:* C1s XPS spectra of all samples. Three different spots per sample were measured to ensure a high reproducibility and the measurements are shown with an y-offset.

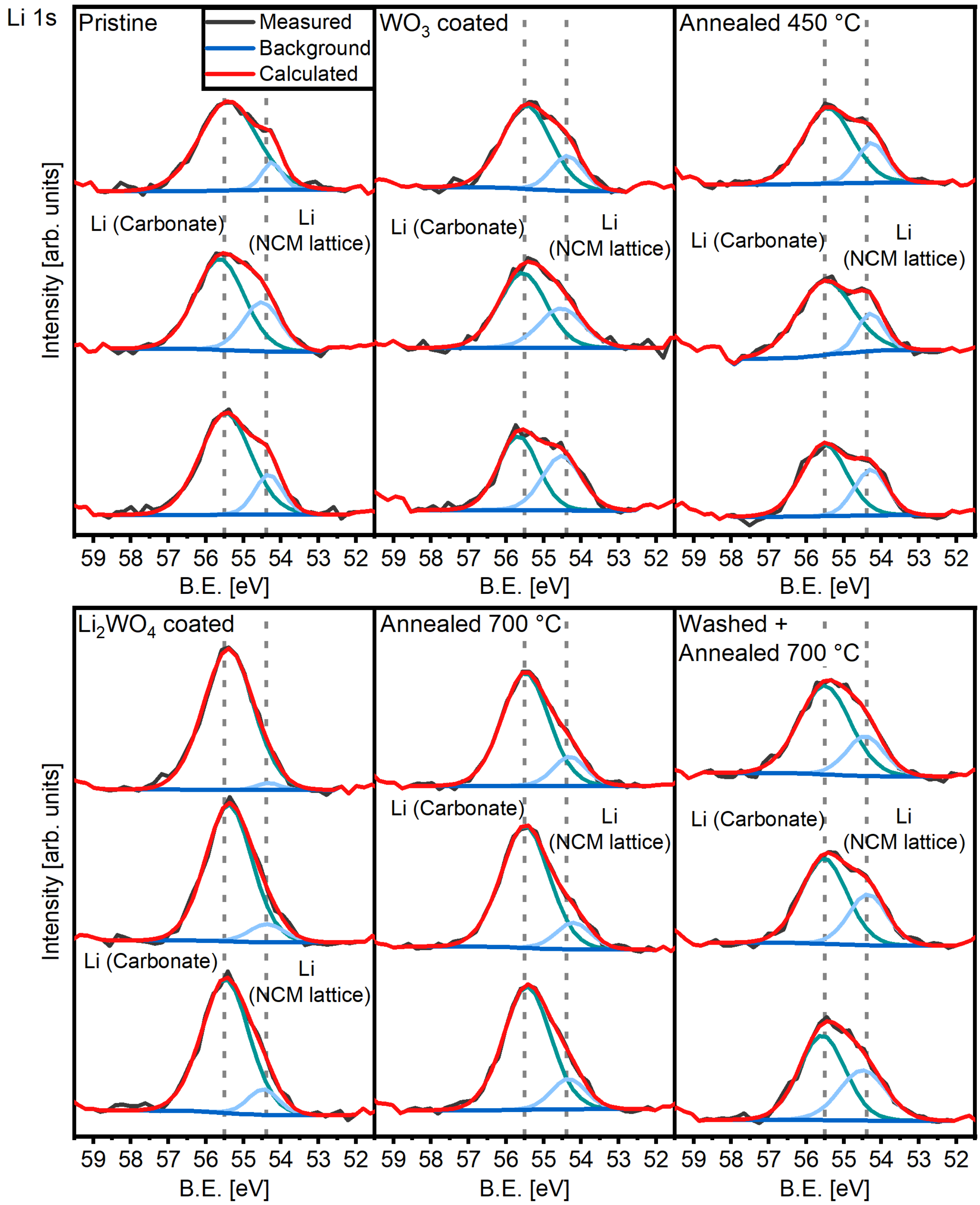

Figure S9: Li1s XPS spectra of all samples. Three different spots per sample were measured to ensure a high reproducibility and the measurements are shown with an y-offset.

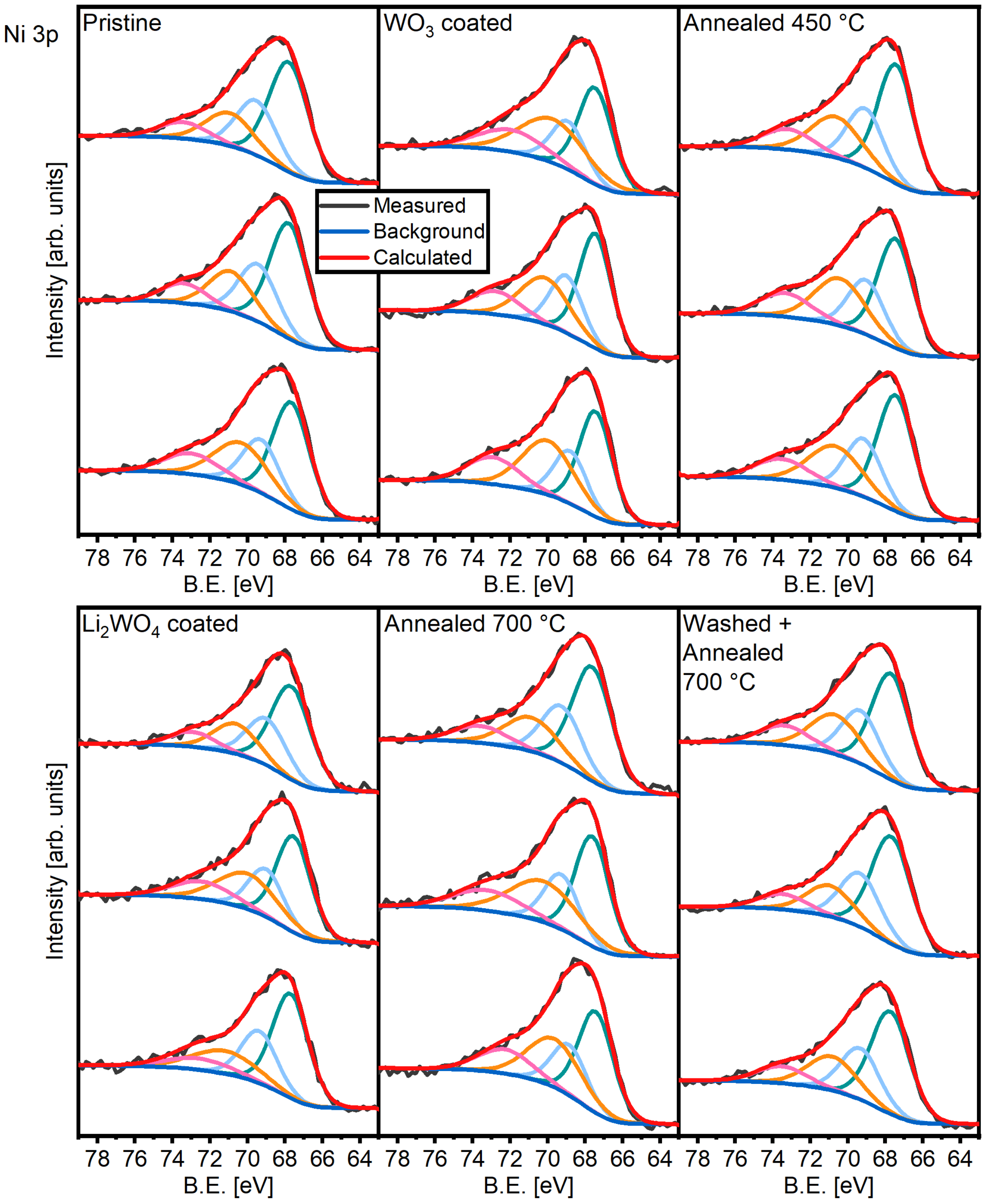

Figure S10. Ni 3p XPS spectra of all samples. Three different spots per sample were measured to ensure a high reproducibility and the measurements are shown with an y-offset.

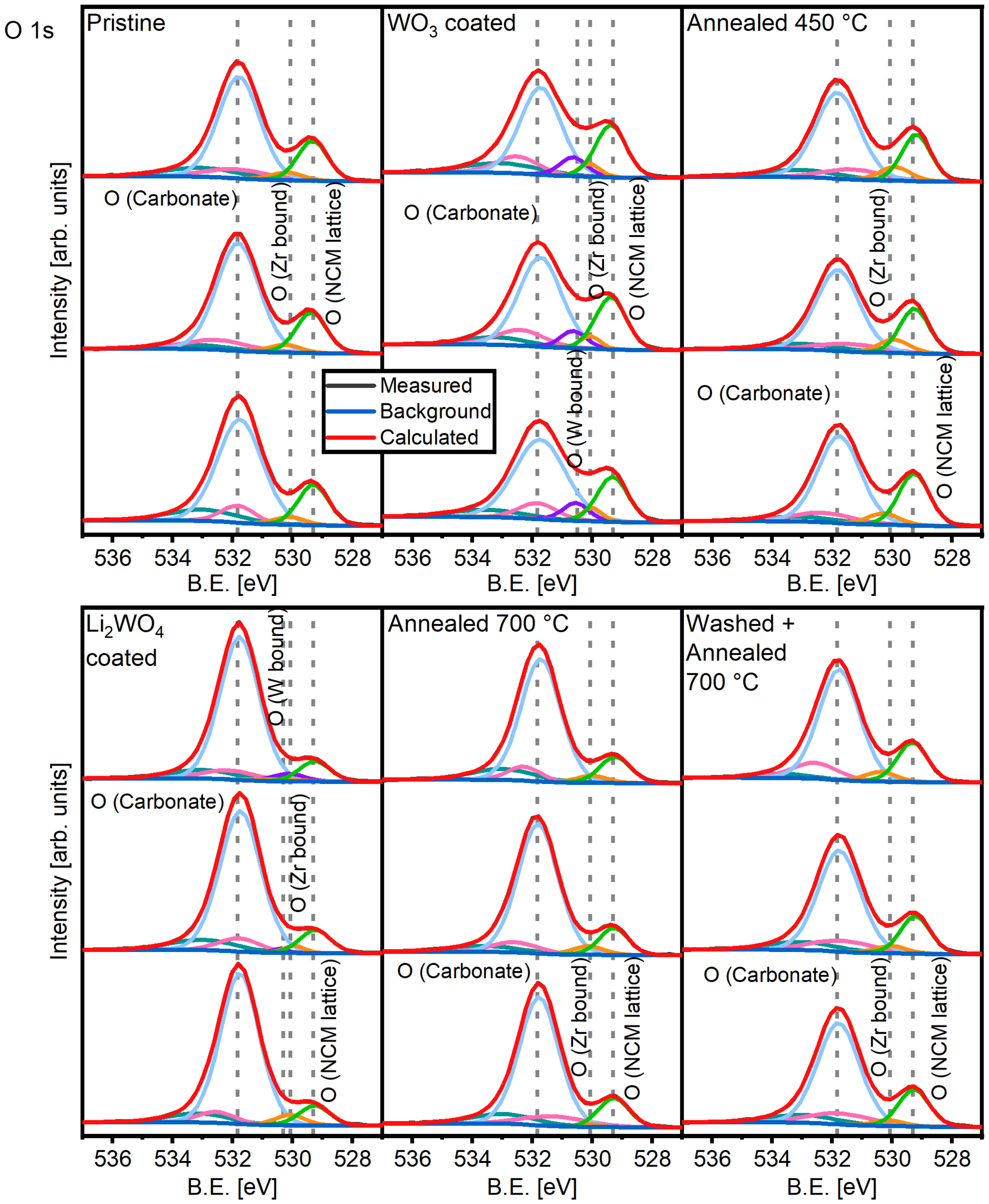

Figure S11. O 1s XPS spectra of all samples. Three different spots per sample were measured to ensure a high reproducibility and the measurements are shown with an y-offset.

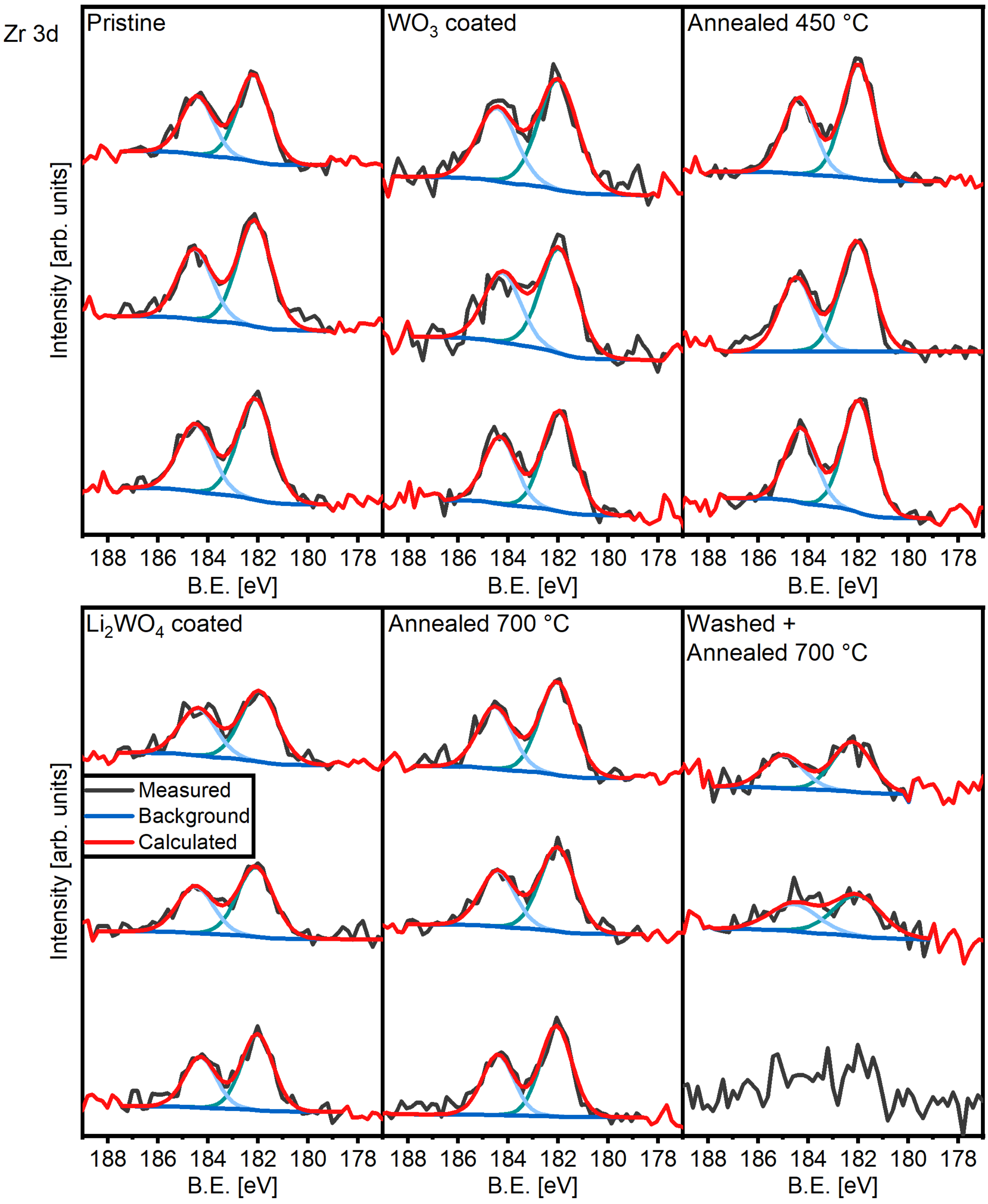

Figure S12. Zr 3d XPS spectra of all samples. Three different spots per sample were measured to ensure a high reproducibility and the measurements are shown with an y-offset. The lowest spectrum of the Li_2WO_4 coated sample could not be reliably fitted and the Zr^{4+} content was considered as 0 for calculating the average.

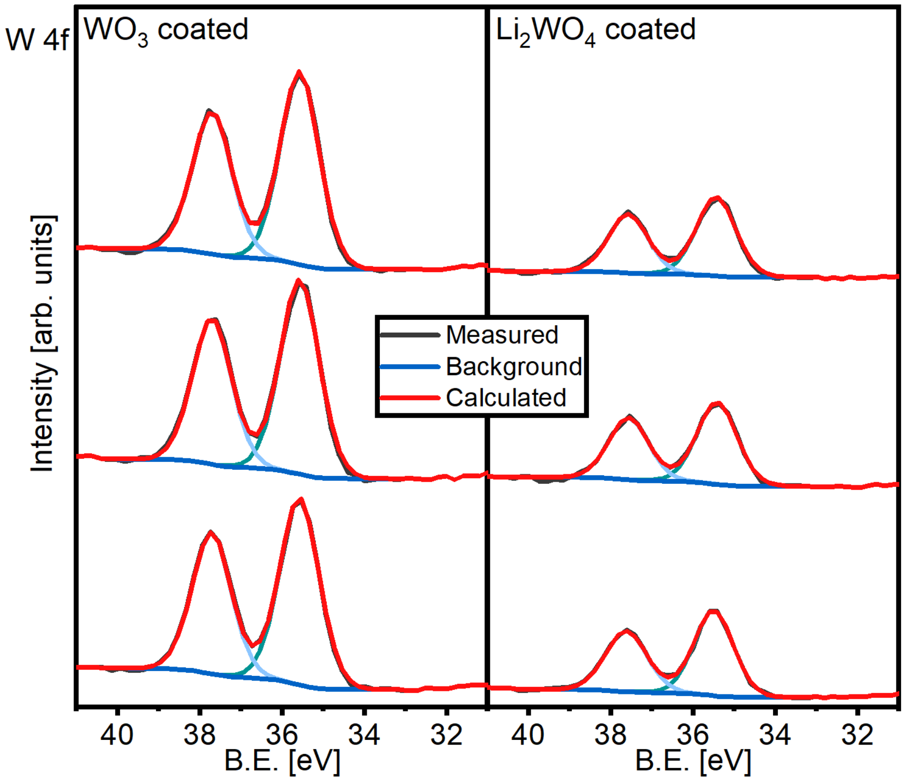

Figure S13. W 4f XPS spectra of the WO_3 coated and the Li_2WO_4 coated sample. Three different spots per sample were measured to ensure a high reproducibility and the measurements are shown with an y-offset.

Electrochemical Characterization

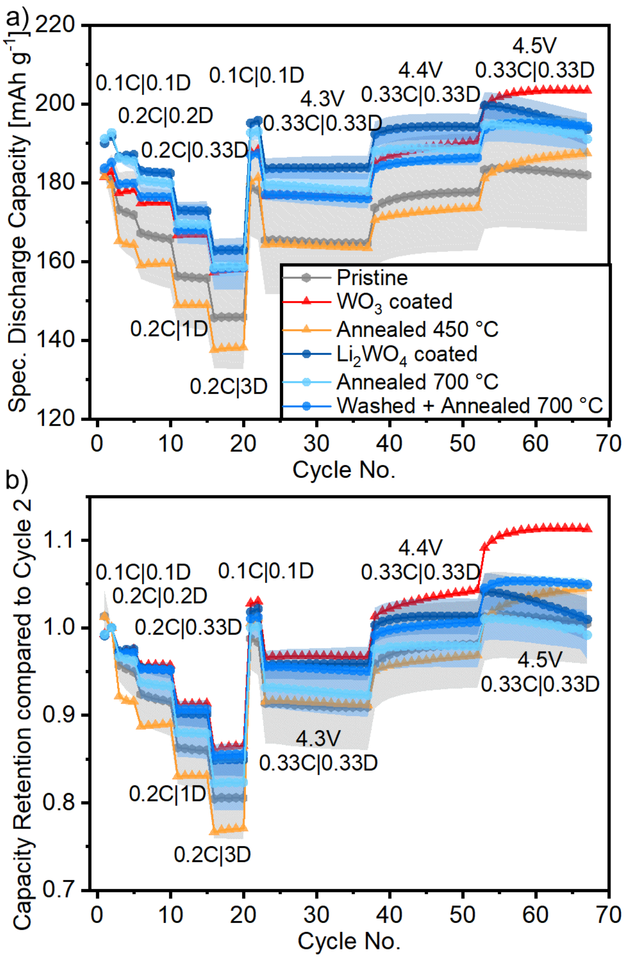

Figure S14. Rate capability investigations in NCM || Li metal cells. a) specific discharge capacity vs. cycle number and b) capacity retention with respect to the 2nd cycle discharge capacity at 0.1C for each material. Cell voltage range: 2.9 - 4.3, 2.9 - 4.4 and 2.9 - 4.5 V. Error bars: standard deviation of three cells for each sample.

Table S4: Results of long-term stability investigation in NCM || graphite full-cells. The given errors are the standard deviation of three cells per sample.

	**Initial Coulombic efficiency [%]**	**Initial discharge capacity at 0.1 C after formation [mAh g^{-1}]**	**Initial discharge capacity at 0.33 C [mAh g^{-1}]**	**End of life (<80% SOH) reached in cycle:**
**Pristine**	80.8 ± 0.4	180.4 ± 0.3	172.6 ± 0.3	343
**WO_3 coated**	81.8 ± 0.1	180.0 ± 2.0	172.0 ± 1.0	730
**Annealed 450 °C**	80.1 ± 0.3	177.5 ± 0.5	168.5 ± 0.4	940
**Li_2WO_4 coated**	84.5 ± 0.3	187.1 ± 0.2	179.3 ± 0.1	521
**Annealed 700 °C**	84.2 ± 0.1	187.1 ± 0.7	179.0 ± 0.8	783
**Washed + Annealed 700 °C**	82.5 ± 0.4	182.8 ± 0.4	175.1 ± 0.2	882

Comparison of pristine and cycled materials *via* SEM

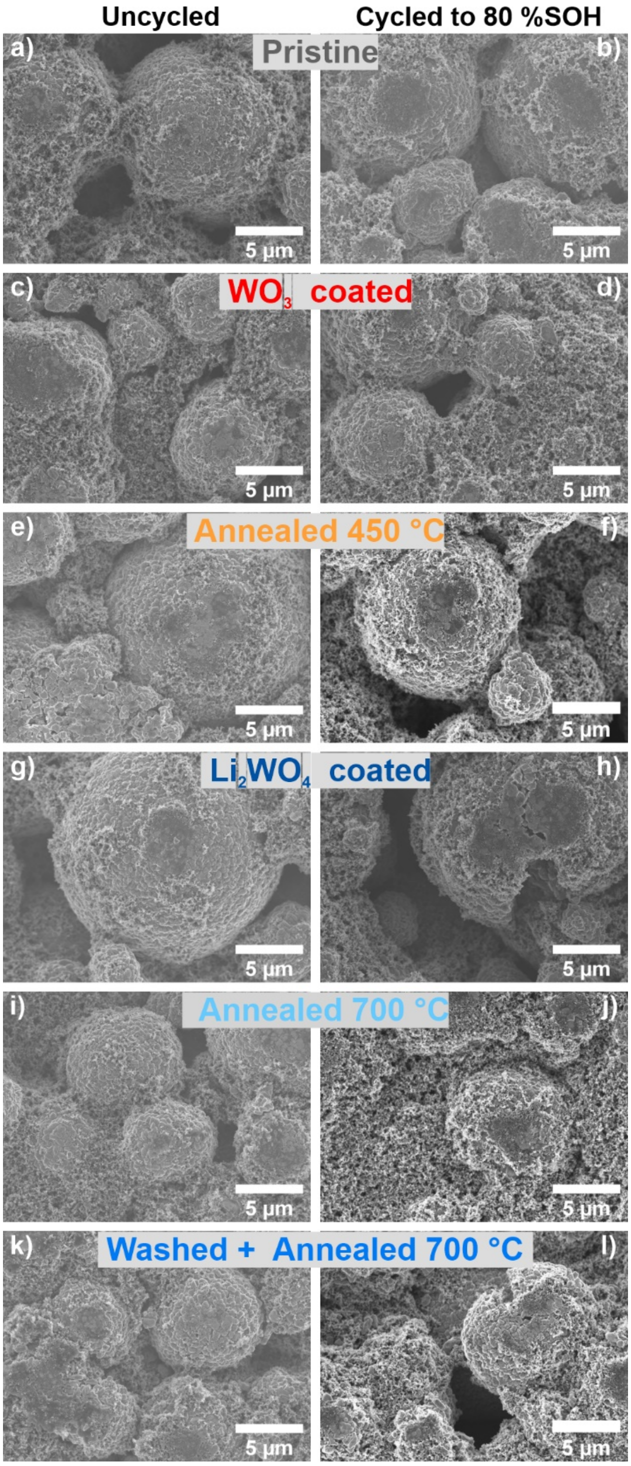

Figure S15. SEM images before and after charge/discharge cycling in NCM || graphite cells until 80% Sate of Health (SOH) (Figure 5) was reached. The number of cycles until reaching 80% SOH is reported in Table S4.
